# Accuracy and quality assessment of 454 GS-FLX Titanium pyrosequencing

**DOI:** 10.1186/1471-2164-12-245

**Published:** 2011-05-19

**Authors:** André Gilles, Emese Meglécz, Nicolas Pech, Stéphanie Ferreira, Thibaut Malausa, Jean-François Martin

**Affiliations:** 1 Aix-Marseille Université, CNRS, IRD, UMR 6116 - IMEP, Equipe Evolution Génome Environnement, Centre Saint-Charles, Case 36, 3 place Victor Hugo, 13331 Marseille Cedex 3, France; 2 Genoscreen, Genomic Platform and R&D, Campus de l'Institut Pasteur, 1 rue du Professeur Calmette, Bâtiment Guérin, 4ème étage, 59000 Lille, France; 3 Institut National de la Recherche Agronomique, UMR 1301, Equipe BPI, 400 route des Chappes, BP 167, 06903 Sophia-Antipolis Cedex, France; 4 UMR CBGP (INRA/IRD/Cirad/Montpellier SupAgro), Campus international de Baillarguet, CS 30016, F-34988 Montferrier-sur-Lez cedex, France

## Abstract

**Background:**

The rapid evolution of 454 GS-FLX sequencing technology has not been accompanied by a reassessment of the quality and accuracy of the sequences obtained. Current strategies for decision-making and error-correction are based on an initial analysis by Huse *et al. *in 2007, for the older GS20 system based on experimental sequences. We analyze here the quality of 454 sequencing data and identify factors playing a role in sequencing error, through the use of an extensive dataset for Roche control DNA fragments.

**Results:**

We obtained a mean error rate for 454 sequences of 1.07%. More importantly, the error rate is not randomly distributed; it occasionally rose to more than 50% in certain positions, and its distribution was linked to several experimental variables. The main factors related to error are the presence of homopolymers, position in the sequence, size of the sequence and spatial localization in PT plates for insertion and deletion errors. These factors can be described by considering seven variables. No single variable can account for the error rate distribution, but most of the variation is explained by the combination of all seven variables.

**Conclusions:**

The pattern identified here calls for the use of internal controls and error-correcting base callers, to correct for errors, when available (e.g. when sequencing amplicons). For shotgun libraries, the use of both sequencing primers and deep coverage, combined with the use of random sequencing primer sites should partly compensate for even high error rates, although it may prove more difficult than previous thought to distinguish between low-frequency alleles and errors.

## Background

Scientific strategies and approaches based on next-generation sequencing (NGS) have been revolutionizing genetics over the last few years. Many aspects of basic, applied and clinical research now rely on the generation of enormous amounts of sequence data from various sample sources, to assess polymorphism (mostly SNPs), or expression data (RNA-Seq) at the genome level [1,2]. This shift in the scale of sequence acquisition has been achieved by simultaneous progress in bioinformatics, the availability of genome assemblies and key technical findings in the domains of biochemistry and sequencing device physics [3]. In this context, the 454 GS-FLX (Roche Diagnostics Corporation), Illumina^© ^technology (Illumina, Inc.) and SOLiDTM systems (Applied BiosystemsTM) offer a number of complementary solutions for specific requirements (see Metzker [4] for a review). 454 GS-FLX Titanium technology provides around 1,000,000 sequences in a single 10-hour run. These sequences, with an average read length equal to 330 bp, may be up to 500 bp in shotgun libraries conditions, much longer than can be obtained with the other available approaches. This makes mapping easier, particularly for repetitive regions, and facilitates *de novo *genome sequencing, exome capture, metagenomics and amplicon sequencing [4].

One of the basic questions arising from this spectacular increase in sequence volume concerns the possible detrimental effects of this shift in quantity on the quality of the obtained data. In other words, is there a tradeoff between the quantity and quality of information? It is widely accepted that next-generation sequencing approaches generate such large amounts of sequence data that even if overall accuracy (derived from error rate) or quality (percentage of error-free sequences) is suboptimal it is still possible to reconstruct polymorphism rigorously by comparing redundant sequences that cover the same genomic region multiple times (i.e. depth of coverage provides accuracy, not the individual read) [5-8]. This is the typical "quick and dirty" view of NGS. This approach may sound reasonable, but it is based on assumptions such as low error rate and error randomness for the unambiguous detection of polymorphism. If this last assumption is challenged, even a low error rate has a huge impact on sequence analysis, as in cases of related allele detection, paralogous sequences or pseudogene identification. In these cases, the "quick and dirty" approach is inadequate, because consensus sequence calculation is accurate only if these three sources of sequence diversity are distinguishable from the error due to background noise [9].

In 2007, S. Huse and collaborators raised the question of the accuracy and quality of massively parallel pyrosequencing GS20 systems, performing an empirical analysis of the per-base error rate [10]. This was needed as "the quality score of a position is not a measure of a confidence that the correct base a called at that position, as with a traditional PHRED score. Instead, the GS20 quality score is a measure of confidence that the homopolymer length at a position is correct" [10,11]. They used V6 hypervariable region sequences from cloned microbial ribosomal DNA for this purpose. They concluded that the accuracy rate was 99.51%, on average, and that 82% of the sequences contained no error. They also demonstrated that 39% of the errors corresponded to homopolymer effects [10]. Finally, they detected no significant correlation between error and distance from the 5' end of the sequences for 101 positions. Surprisingly, despite changes in this technology over the last four years, the accuracy and quality of 454-based sequences has not been reevaluated and this previous study remains the basic reference used by the scientific community to account for error rate in 454 GS-FLX systems (181 articles citing this study at the time of writing). Over the same period, chemistry, acquisition devices (CCD cameras in particular) and quality filtering algorithms have evolved. A new analysis is therefore required, and this was the main goal of this work.

Furthermore, in addition to estimating the per-base error rate, we aimed to identify the potential causes of sequencing errors and possible solutions for improving both the accuracy and quality of pyrosequences. We selected several variables likely to affect sequencing errors directly or indirectly: (i) the position of the nucleotide base within the sequence (the beginning of the sequence may be more accurate than the end), (ii) the primary structure of the sequence, including, in particular, the presence of homopolymers, (iii) the length of the sequence generated (a sequence may be short due to quality filtering, resulting from an accumulation of errors or the stochastic ending of polymerization), and (iv) the position of the bead carrying the sequence both within and between the regions on a PT plate (PicoTiterPlate) (edge effect), and between multiple PT plates. Our analyses are based on Roche test fragments. These are sequences used for GS-FLX Titanium diagnostics that are included in all runs, but not subjected to PCR amplification before sequencing. Thus with these fragments we estimate the sequencing error due to pyrosequencing. Huse et al. [10] found that the experimental sequences they used display error rate five times higher than the GS20 Roche test fragments (0.1% vs. 0.49%). Since almost all of their results are based on experimental sequences, we cannot directly compare our results to theirs. However, we do not intend to focus on a general error rate, but rather assess the effect of several variables on error generation.

## Results and Discussion

### Accuracy and quality of sequences

We assessed the quality of the sequences obtained by 454 GS-FLX Titanium sequencing, using the control DNA fragment Type I sequences (provided with 454 sequencing kits) as reference templates (see Materials and Methods for details). As these internal controls are added to the pyrosequencing process during the sequencing step, they are modified only by sequencing errors and are not related to any previous step. The quality of these control sequences is not influenced by the samples themselves, particularly with Titanium technology, in which loading beads are isolated from each other and there should therefore be no interference from adjacent beads. We analyze here the 86,237 sequences that passed the quality filters, representing the six control DNA fragments from three 454 GS-FLX runs. These results revealed several general trends in the sequencing error generated by 454 GS-FLX Titanium technology (Table 1). It also provided detailed information about the different types of error: insertion, deletion, mismatches and ambiguous base calls. We first analyzed the error on the first 101 sequenced positions from the 5' end (with reference to the sequencing primer) of the control DNA fragments. We compared the sequences obtained with those for the GS20 system and then extended the error analysis to full-length sequences (500 to 592 bases, depending on the reference sequence analyzed, see Materials and Methods for details).

**Table 1:** Comparative analysis of the accuracy and quality of sequences

	# of sequences	% of error-free sequences	# of positions	Insertions	Deletions	Mismatch	Ambiguous	Total % of error
	
GS20 (101)	34015	82.00%	32801429	0.18%	0.13%	0.08%	0.10%	0.49%
Ref 1 (101)	16052	87.12%	1605640	0.15%	0.05%	0.01%	0.01%	0.22%
Ref 2 (101)	16466	60.01%	1600327	0.42%	0.23%	0.04%	0.01%	0.70%
Ref 3 (101)	12215	72.96%	1228804	0.17%	0.19%	0.01%	0.01%	0.38%
Ref 4 (101)	9908	56.43%	984452	0.30%	0.37%	0.03%	0.00%	0.70%
Ref 5 (101)	15880	50.93%	1595718	0.34%	0.48%	0.05%	0.01%	0.88%
Ref 6 (101)	15716	75.17%	1581075	0.25%	0.10%	0.00%	0.01%	0.36%
Total	86237	67.57%	8596016	0.27%	0.23%	0.02%	0.01%	0.53%

Ref 1 (572)	16052	6.75%	5359696	0.52%	0.46%	0.10%	0.12%	1.20%
Ref 2 (552)	16466	9.75%	4789285	0.89%	0.28%	0.10%	0.08%	1.35%
Ref 3 (500)	12215	18.75%	4180478	0.30%	0.35%	0.07%	0.12%	0.84%
Ref 4 (532)	9908	6.88%	2572843	0.56%	0.71%	0.19%	0.11%	1.57%
Ref 5 (592)	15880	7.46%	6171098	0.38%	0.38%	0.06%	0.07%	0.89%
Ref 6 (516)	15716	11.81%	6027338	0.60%	0.17%	0.07%	0.04%	0.88%
Total	86237	10.09%	29100738	0.54%	0.36%	0.09%	0.09%	1.07%

The different types of error are detailed for each reference sequence for 454 sequencing. Errors are classified according to the nomenclature used by Huse et al. (2007): insertions, deletions, mismatches and ambiguous base calls (see materials and methods). Error rates are given for two length categories (first 101 bases vs. full length).

The error rate for the first 101 sequenced positions (corresponding to 8,596,016 examined bases) displayed a mean = 0.534% (95% CI: [0.529, 0.539]) (45,895 erroneous bases) for 454 GS-FLX Titanium data. This global error rate is five times higher than the error rate obtained by the analyses of GS20 test fragments and is similar to that obtained from for GS20 experimental sequences. Indeed, 0.49% of the positions were erroneous for a comparable dataset relating to 101 positions (Table 1). If we break down the global error rate for all reference sequences according to the type of error, insertions are found to be the most common errors (mean = 0.273% [0.269, 0.276]; mode q_1/2 _= 0.215), followed by deletions (0.232% [0.229, 0.235]; q_1/2 _= 0.170), mismatches (0.022% [0.021, 0.023]; q_1/2 _= 0.010), and ambiguous base calls (0.007% [0.006, 0.007]; q_1/2 _= 0.010). This pattern is entirely consistent with that described by Huse *et al. *[10]. This pattern is in agreement with the study of 454 GS-FLX [12] but markedly different from IlluminaTM sequencing, in which insertions and deletions of single bases occur less frequently than mismatches [13,14]. In total, 58,269 sequences (67.57% [67.26, 67.88]) of this length were found to be free from error. This trend is similar to that reported for GS20 experimental sequences, for which 82% of sequences matched the corresponding reference sequence perfectly. Unfortunately the data are not available for GS20 test fragments.

If we restricted the analysis to full-length sequences (500 to 592 positions), we found for the 86,237 sequences that passed the 454 quality filters (29,100,738 bases) that 312,351 bases were erroneous (1.073% [1.069, 1.077]). The pattern observed for the first 101 positions was confirmed for the full-length sequence data, with insertions (0.541% [0.538, 0.543]; q_1/2 _= 0.465) and deletions (0.359% [0.357, 0.362]; q_1/2 _= 0.350) being the most common types of error and mismatches (0.088% [0.087, 0.089]; q_1/2 _= 0.085) and ambiguous base calls (0.085% [0.084, 0.086]; q_1/2 _= 0.090) making a smaller contribution to global error rate. Only 8,702 of the 86,237 full-length sequences (10.09% [9.89, 10.29]) had no error with respect to the corresponding reference sequence. This result strongly contrasts with the higher proportion of error-free sequences for the first 101 bases.

The comparison of error rates between sequences of different lengths (first 101 positions *vs *full-length sequences) highlighted two key developments in addition to the doubling of the global error rate for full-length sequences as found elsewhere [15]. This length-associated overall increase in error rates did not reflect a common mechanism for all types of error, as insertion and deletion rates increased only slightly (by factors of 2 and 1.5, respectively), whereas mismatch and ambiguous base call rates increased to a much greater extent (by factors of 5 and 9, respectively). This decoupling of the changes in rate for different types of errors modified the contribution to global error rate of the various types of errors. Thus, mismatch and ambiguous base call errors made a greater contribution to global error rate for longer sequences, although their effects remained moderate. Thus, overall error rates and the rates of different types of error are not uniform for the sequences obtained by 454 GS-FLX Titanium sequencing. Consequently, the conclusions drawn for short sequences should not be directly extrapolated to longer sequences, as sequence length affects error rates. Another key result in this in-depth analysis of error was the finding that error rate (1.07%) should be seen in the light of the large number of erroneous sequences (89.91%) in the dataset. This combination of a low error rate and a large number of erroneous sequences results from the occurrence of only very small numbers of errors in individual sequences, on average. These findings conflict with those reported for GS20 sequencing and suggest that the removal of erroneous sequences may not be useful, to increase the overall quality.

**Figure 1:**
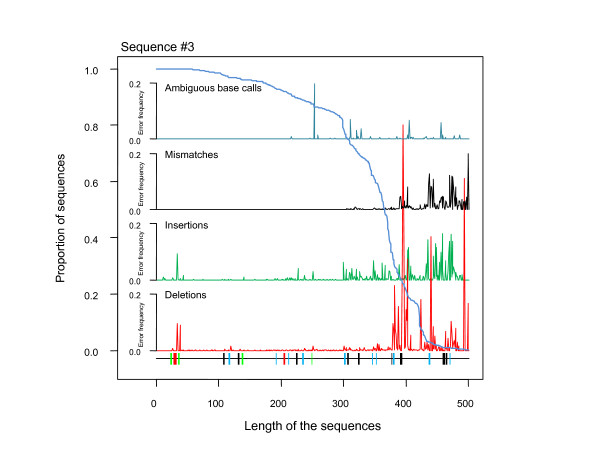
**Distribution of errors along sequences**. The blue line indicates the proportion of generated sequences (y-axis) as a function of sequence position (x-axis), based on data obtained from the analysis of reference sequence #3. The error rate for each type of error (insertions, deletions, mismatches and ambiguous base calls) is presented as a function of sequence position (X-axis) and specific position on the y axis. The position and length of homopolymers for each base are given on the x-axis to facilitate interpretation (green: A, red: T, black: G, blue: C). See additional file 2 for the data obtained with the other five reference sequences.

However, the consequences of this may be relatively minor even if most sequences display errors (89.91 [89.71, 90.11]), as the overall error rate is low, with only 1.07% of bases being problematic. It is widely believed that deep sequencing coverage (multiple independent sequences for the same locus) should make it possible to correct for errors in this context [16]. Like other types of high-throughput sequencing, 454 pyrosequencing is thought to be suitable for use in this context. Indeed, for some applications, such as SNP discovery in whole-genome sequences [17,18] or amplicon sequencing [7,19], an almost unlimited number of sequences may be obtained. We need to consider the number of sequences required to correct an erroneous position appropriate, at a given probability, for an error rate of 1.07%. As detailed in additional file 1, at least five sequences would be required to correct for random error at low error rates (<10% error rate). However, an analysis of error along the length of the sequence (comparing the first 101 bases with the full-length sequence) indicated that error rate was heterogeneous along the length of the sequence. Longer sequences therefore would be subject to higher error rates at their 3' ends. The distribution of error, as illustrated in Figure 1, does not fit a stochastic model, for any error type. Most of the positions are correct, but a few have high error rates, even exceeding 50% in some cases. There is no clear way to resolve the issue, particularly when this pattern (error hot spots) is repeatable between runs [9].

This pattern is particularly problematic for 454 data, as the number of sequences significantly decreases after 300 bases (see Figure 1 and additional file 2 for illustration) whatever the reference sequence considered (for a total length ranging from 500 to 592). In summary, for the longest sequences (>300 positions), the combination of higher error rates along the length of the sequence, combined with the decrease in the number of sequences available, may make it difficult to correct errors. This difficulty results from a deficit in the number of sequences required to decrease the probability of erroneous assignation for a given sequence position, under a reasonable coverage threshold (i.e. minimum number of reads per bp required, see additional file 1).

This issue is further complicated by the heterogeneous distribution of the error types among the six different control DNA reference sequences, within and between gasket regions for a PT GS-FLX Titanium plate and also between PT plates, as initially estimated from the large standard errors (derived from table 1) in the error estimate. This overall variability of error distribution makes it difficult to draw any clear conclusions ruling out particular parameters that might potentially influence error rates or to identify a single mechanism accounting for the observed errors in the dataset. This pattern requires an in-depth analysis of the interaction and explanatory power of various factors before we can assess the degree of sequencing error and identify solutions for preventing artifacts.

### Interactions between variables and error characterization

The evolution of 454 technology combines progress in chemistry, acquisition devices, such as CCD cameras and PT plates handling equipment, and improvements in quality filters and base-calling algorithms. All these modifications are potential sources of variation in the amount, length and quality of sequences. In this work, we analyzed the interaction of seven variables identified as potential sources of sequencing error. We characterized sequencing error as a function of information about position in the sequence (*Position *and *Seq.length*), the presence of homopolymers (*Homopolymer*) and reference sequence type (*Seq.type*), all considered being sequence-specific information. Location on the PT plate was also taken into account through the region of origin (*Region*), the distance of beads to the region center (*Dist.region*) or the plate center (*Dist.plate*, see Materials ad Methods for details) as both the flow of chemicals through the plate and the central position of the CCD camera may play a role in the error generation. Before this analysis, we tested the hypothesis of homogeneous error rates on the three PT plates. This hypothesis was rejected (χ^2 ^= 2613.3, df = 2, P < 2 × 10^-16^). The significant result obtained in this test is mostly due to the high power of detection associated with the large number of samples available, but this heterogeneity requires the specification of individual parameter values for the logistic model describing each PT plate. The three runs were therefore analyzed separately. This approach did not prevent us from extracting the common trends influencing error rate and distribution. The models (for each plate and for each type of error) explained between 14.32% and 37.38% of the error distribution and were highly significant (P < 2 × 10^-16^).

The nullity of r (Bravais-Pearson correlation coefficient) between pairs of the seven variables was tested independently for each run. As the usual assumptions required to infer the distribution of the test statistics were not met, we used permutations to approximate the distribution of the test statistic under H_0_. We used a type I error rate of 0.05 and Benjamini-Hochberg correction [20] to take multiple testing into account. Most of the pairs of variables (74.29%) were significantly correlated, using a threshold of α = 0.05 in a permutation test for multiple testing. However 41.85% of the pairs of variables correlated with 0.005 < r < 0.05. The pair of variables displaying the strongest correlation was the position of the error in the reference sequence (*position*) and sequence length (*Seq.length*), with 0.40 < r < 0.50, depending on the PT plate considered. The second strongest correlation was that between distance to the region center (*Dist.region*) and distance to the PT plate center (*Dist.plate*), with 0.38 < r < 0.62.

**Figure 2:**
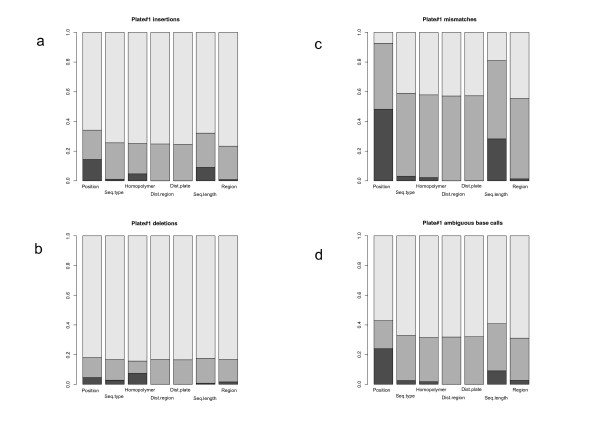
**Decomposition of error rate variation, using all available variables**. For each plate, we used a logistic model to decipher the role of each selected variable and its contribution to error rate (see materials and methods). The error rate has been broken down as a function of error type: a) insertions, b) deletions, c) mismatches and d) ambiguous base calls. We tested the deviance from the complete model by breaking down the complete model into the sum of three terms: the first exclusive to the single effect of the variable considered (in black), the second exclusive effect of the rest of the variables without the variable of interest (in gray) and the last expressing the sum of the effects of interactions between the variable considered and the other variables (in white). The contribution of each term (the proportion) for a considered variable can be viewed on the y-axis. We display only the results for plate #1 (the results for the other plates are presented in additional file 3).

The nature and significance of a correlation between two variables does not provide any information about the ability of this combination of variables to explain a third variable [21]. For each plate and each kind of error, we considered a logistic model [22] (see materials and methods for the detailed procedure) accounting for the binary (error) variable in terms of the seven variables considered. For the separation of the effect of a given explicative variable from the combined effect of the other variables, we propose (see materials and methods) breaking down each explanatory variable into three additive terms: the effect of the variable itself, the combined effect of the other variables and the rest. The combined effect of the variables ranged from 20% to 80% of the total variation in error rate (Figure 2 and additional file 3). More specifically, for individual error types, the combined effect accounted for 38.00% ± 13.05 of the total information for mismatch errors, 64.10% ± 4.54 for ambiguous base call errors, 75.83% ± 3.78 for insertion errors and 79.95% ± 3.08 for deletion errors. The remaining information results from the specific effects of each variable. These high percentages of shared information highlight the high degree to which the error can be explained by combinations of variables. This may be due to partial redundancy of the information contained in each variable or the combined contribution to the total amount of error explained [21]. In the first case, a variable may substitute for the effect of others, whereas, in the second, only the combined information provided by each variable can account for the observed pattern. The results of correlation analysis, indicating that most regression coefficients were low, ruled out redundancy as the primary cause of the observed pattern, as most variables were independent. There is therefore no single variable consistently accounting for the distribution of sequencing error, as detailed in Figure 2. We investigated the main trends highlighted by the logistic model, by focusing on the distribution of sequencing error at sequence level. We then characterized the variables most strongly influencing error in terms of the location of the bead carrying the sequence, in a given region of a PT plate.

At DNA sequence level, we detailed the variables individually accounting for the highest proportion of the error rate for each error type. It was essential to bear in mind, during this analysis, the fact that most of the explanatory power of these variables was obtained with combinations of variables. We analyzed each type of error independently.

For insertion errors (Figure 2), the variable *Homopolymer *accounted for 5.97% ± 1.33 of the variation in error on its own, and was concurrent to the error rate. This finding is consistent with available published empirical observations linking errors to homopolymers [9]. The variable *Position *accounted for 11.94% ± 2.22 of the variation and was also concurrent to error. In other words, the error rate due to insertions increased along the sequence. Finally, the variable *Seq.length *accounted for 5.48% ± 3.13 of the variation. Insertion rates were lower for longer sequences and higher for shorter sequences. These last two results may appear paradoxical, but the combined information for these variables indicates that the distribution of insertion errors along sequences is not random, with more insertions in 3' end, whatever the length of the sequence considered. This is fully explained if we considered that i) the number of sequences decreases with length (Figure 1), hence changing the number of sequences for which error rates are computed with respect to the reference and ii) the quality filtering process (v2.3) implemented in the GS-FLX system involves the trimming of reads with many off-peak signal intensities by the software. In particular, for insertions, the TrimBack Valley Filter trims sequences from the 3' end until the number of valley flows (intermediate signal intensity, i.e., a signal intensity occurring in the valley between the peaks for 1-mer and 2-mer incorporations, or 2-mer and 3-mer, etc.) is < 1.25% [23]. This implies that short sequences are not short because the strand synthesis stops prematurely, but due to a rapid decrease in the quality of the flowgram (raw sequence) resulting from early out-of-phase synthesis. Trimming eliminates the 3' end with above-threshold ambiguous base calls, but the remaining sequence still contains errors.

For deletion errors, *Seq.type *accounted for 2.36% ± 1.39 of the variation, reflecting substantial heterogeneity between the reference sequences. The variables *Homopolymer *(accounting for 6.89% ± 0.89 of the variation) and *Position *(accounting for 8.93% ± 5.21 of the variation) were both concurrent to the deletion rate. Deletion errors tend to occur more frequently in homopolymers and their rates are higher towards the 3' end of sequences.

Finally, mismatch and ambiguous base call error rates were both found to be linked to *Position *(45.24% ± 4.04 and 25.85% ± 1.71, respectively) and *Seq.length *(25.00% ± 9.61 and 7.66% ± 2.11, respectively), with higher error rates found in 3' positions within sequences and longer sequences tending to have lower error rates.

**Figure 3:**
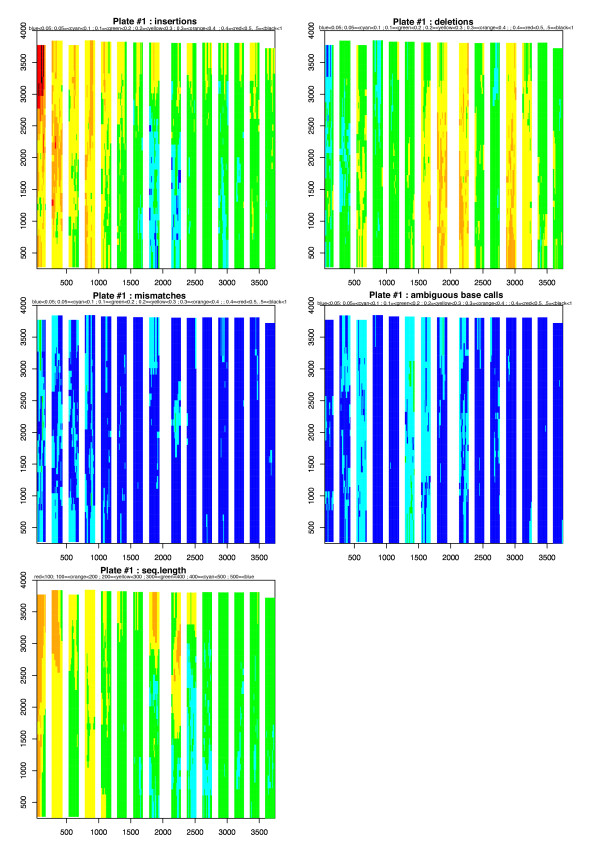
**Spatial distribution of error rate variation**. For each error type and sequence length, the x-axis represents the spatial location of 454 reads and the y-axis represents the y-coordinates on the PT plate. The results presented in this figure correspond to plate #1. Data for the other two runs is presented in additional file 4. The 15 strips represent the 15 regions. We display separately the four types of error (insertions, deletions, mismatches and ambiguous base calls) and the length of the sequences generated. Colors indicate the ranges of error rates, from 0 to 1 (or the length of the sequences, from 0 to 500), using a sliding window (see materials and methods).

Given this pattern, the next step in the integration of information is characterizing the effect of bead localization on error rate. In particular, it is useful to consider whether position in a particular region or on the PT plate is linked to error rate. Heterogeneity in error rate as a function of bead location was found for insertions and deletions, whatever the PT plate analyzed. Heterogeneity was observed at both the region and plate scales. More precisely, error rate variation was mostly accounted for by the combination of several variables but, when the distribution of insertion errors fitted a gradient following the Y-axis in each region (Figure 3 and additional file 4), it was not accounted for by the variable *Dist.region *alone. However, the proportion of the model accounted for by the remaining variables is small (23.01% ± 2.62). Adding the *Dist.region *to the model increases explanatory power to 76.99% ± 2.62. The situation was similar for extraction of the signal at plate level, with *Dist.plate *increasing the explanatory power to 77.39% ± 2.12. In summary, all regions had heterogeneous insertion and deletion error rates, but there were conserved gradients along both the x and y axes. Inverse physical gradients were observed for insertions and deletions. The covariation of these error types and sequence length indicates that they are influenced by a single latent variable (Figure 3).

## Conclusions

### From statistical inference to technical causes and perspectives

As detailed in the results and discussion section, error rate variability is mostly accounted for by the combination of the seven variables analyzed. However, the heterogeneous physical pattern may be partially driven by the combined influence of the central CCD camera (edge effect) with chemical flow direction (Y-axis). This explanation is, however, insufficient in itself to account for the observed pattern, and other variables clearly influence error rate. The negative relationship between insertion and deletion errors is probably related to physical acquisition issues, but chemistry-related artifacts probably also have an effect (through the related statistical variables analyzed), including the CAFIE effect (carry forward and incomplete extension) in particular. Carry forward occurs when a trace amount of nucleotide remains in a well after the apyrase wash, perpetuating premature nucleotide incorporations for specific sequence combinations during the next base flow and contributing to signal 'noise'. Incomplete extension occurs when some DNA strands on a bead fail to incorporate during the appropriate base flow. The strands that fail to incorporate must await another flow cycle for sequencing to continue and are thus incorporated out-of-phase with the rest of the strands [23].

This study clearly demonstrates that sequencing error rate, as deciphered here, is a heterogeneous feature in 454 GS-FLX Titanium pyrosequencing. We cannot extrapolate the results obtained for other technologies, such as the GS20 system, to this system, nor is the use of a single global error rate inappropriate. Our results provide information about the number of sequences required to correct for a specific erroneous position, when detected, but this procedure requires the error rate to be computed from within the 454 PT plate regions in which the physical distribution of error rate is heterogeneous. Internal DNA controls should therefore be used when appropriate [7,19,24] (readily available for amplicon sequencing), together with an error-corrected base caller [25], and routine procedures taking error data into account should be defined. When error rate is not estimated, a large number of potential false-positive polymorphisms would be expected and only post-sequencing validation can account for these artifacts [26,27]. For the resolution of this issue, the use of both sequencing primers and deep coverage, combined with the use of random sequencing priming sites, should partially compensate for error -- even for high error rates -- although it may be more difficult to distinguish between low-frequency alleles and errors than previously anticipated.

## Methods

### Experimental design and reference sequences

We used the six control DNA fragment Type I sequences (as provided in Roche 454 protocols) as reference sequences. This made it possible to use a large number of strictly identical templates to characterize the sequencing error rate of this technology. The sequences generated constituted a set of three replicates from three different runs, making it possible to assess the quality and accuracy of the 454 GS-FLX Titanium method. Six references were used, with lengths ranging from 500 to 592 bp and GC contents from 52.75% to 65.85%; each of these reference sequences contained a large number of homopolymers (20 to 34), defined as a succession of three or more identical bases. Homopolymer positions are shown on Figure 1 and in additional file 2. The reference sequences are provided in additional file 5.

All reference sequence positions were classified according to the presence and length of a homopolymer: (i) the first and last bases of a homopolymer and those within two bases on either side of a homopolymer were coded "1". All the other positions within the homopolymer were coded "3" to "6" (the length of the homopolymer). All positions outside these zones (not influenced by the homopolymer) were coded "0".

The dataset consisted of 86,237 sequences, corresponding to 29,100,738 positions. Sequencing was carried out at Genoscreen, France. We aimed to identify factors linked to error rate. For a tractable analysis, we analyzed a dataset corresponding to all the positions at which an error was detected, plus a similar number of error-free positions randomly selected from the whole original dataset.

### Sequencing error analysis

Reads (see additional file 6) were sorted according to their reference sequences, by BLASTn [28]. Each read was aligned to its reference sequence, to identify the positions and the number of sequencing errors. For optimization of the pairwise alignment parameters, the total number of errors was counted in a test dataset of 500 kb for a series of gap opening and gap extension penalties. The final analyses were carried out with ClustalW [29], using "1" as the gap opening penalty, and "10" as the gap extension penalty.

In the analyses, the observation unit was the position on the 454-generated sequences. These positions were transformed into the position on the reference sequence. Insertions are reported with respect to the position of the base preceding the gaps. For each position, a binary variable was defined indicating the presence or absence of a sequencing error. An error is defined here as discordance between two homologous positions: the first in the reference sequence and the second in the generated sequence. Discordance may refer to an insertion, a deletion, a nucleotide mismatch or an ambiguous base call (N) with respect to a non-available nucleotide determination on the replicate sequence (according to Huse *et al. *[10]). We investigated the pattern of 454-error type, focusing on the following seven factors: (i) ***Position***, position in the sequence expressed as a proportion of the total length of the reference sequence (treated as a quantitative variable); (ii) ***Seq.type***, the different reference sequences (qualitative variable with 6 settings); (iii) ***Homopolymer***, type of homopolymer linked to the position as defined above; (iv) ***Dist.region***, Euclidean distance between the generated sequence (bead) and the center of the region on the plate; (v) ***Dist.plate***, Euclidean distance between the generated sequence and the center of the plate; (vi) ***Seq.length***, length of the considered generated sequence (the observed sequence length results from the GS-FLX quality filtering process); (vii) ***Region***, region of the plate in which the replicate was observed, region of the considered replicate.

The R package was used for all statistical tests [30]. The significance of regression coefficients was assessed by a permutation test with Benjamini-Hochberg correction, with α = 0.05. As we studied both qualitative and quantitative variables, we decided to transform the qualitative variables. The various possible settings of each qualitative variable were therefore replaced by a binary variable (dummy variable).

Let us define as π*i *the sequencing error rate for the position *i*. As this value is supposed to vary as a function of the factors defined above, we have π*I = P(Y*_*i *_= 1/x^*^_i_*) = E(Y*_*i*_/x^*^_i_*) = *π(x^*^_i_). *Y*_*i *_is the binary variable equal to 1 if an error is present and 0 otherwise. x^*^_i _is the vector (*x*_*1i*_, *x*_*2i*_, ..., *x*_*7i*_) of the explanatory variables. We chose to model the error rate π(x^*^_i_) with a logistic model [22]:

Maximum likelihood estimators were considered to estimate the parameters of the model. Tests of significance of the parameters were then carried out with Student's t test. A model was generated for each of the three plates and for each of the error types (insertion, deletion, mismatch and N). All the analyses were performed with R (version 2.6.0).

The contribution of a given explanatory variable *xi *is assessed as follows. Let us denote by *comp.mod *the logistic model including all the variables considered, and *dev(comp.mod)*, its deviance. Let us define *dev(sub.model) *as the deviance associated with the model including all the variables other than the considered *xi*. Then, *part(xi) = (dev(sub.mod)- dev(comp.mod))/dev(comp.mod)) *expresses the contribution of *xi *in addition to the other variables. We can symmetrically define the participation of all the variables other than *xi: part(whole\xi) = (dev(xi)-dev(comp.mod))/(dev(comp.mod))*. Hence the deviance of the complete model may be broken down into the sum of three terms: the first exclusive to *xi*, the second exclusive to the rest of the variables and the last expressing the explanation common to *xi *and the other variables: *1 = part(xi) + part(whole\xi) + (1- part(xi) - part(whole\xi))*.

## Authors' contributions

AG conceived the study and wrote the manuscript. EM participated in the design of the study, performed the bioinformatics analysis and helped to write the manuscript. NP participated in the design of the study, performed the statistical analysis and helped to write the manuscript. SF participated in the design and performed the molecular biology. TM helped to write the manuscript. JFM conceived the study and wrote the manuscript. All authors have read and approved the final manuscript.

## Supplementary Material

Additional file 1 **Number of sequences to correct erroneous positions**. 1a: this file illustrates the number of sequences necessary to obtain a majority of correct sequences. The x-axis shows the error rate and the y-axis shows the number of sequences needed, according to three possible probabilities: 0.001 0.01 and 0.05. 1b the x-axis shows the error rate for a given position (ranging from 0 to 0.5); the y-axis shows the cumulative proportion of erroneous sequences sampled (ranging from 0 to 0.5) in the total sample. Sample size varies from 10 to 100, 500 and 1,000 sequences. For a given error rate and a cumulative proportion of erroneous sequences in the sample of size N, the probability of observing this combination is indicated in color: green: 1 to 0.95, blue: 0.95 to 0.8, yellow: 0.8 to 0.6, orange: 0.6 to 0.5, red: 0.5 to 0.4, gray: 0.4 to 0.2 and white: below 0.2. For example, if the error rate is 0.2, the probability of observing a cumulative proportion of erroneous sequences in the sample of between 0 and 0.2 ranges between 0.4 and 0.5 (red envelope). In this case, the probability of there being 20% erroneous sequences in the sample is between 0.4 and 0.5. If we consider the same error rate (0.2) with 40% erroneous sequences, then the probability ranges from 0.8 to 0.95 (blue envelope). If N increases, the variance of the probability envelopes decreases. Click here for file

Additional file 2 **Distribution of errors along the reference sequences**. The blue line represents the proportion of sequences generated (y-axis) according to the sequence position (x-axis), using data obtained from the analysis of reference 5 reference sequences (excluding reference #3, which is displayed in Figure 1). The error rate for each type of error (insertions, deletions, mismatches and ambiguous base calls) is presented as a function of the sequence position (x-axis) and specific position on the y-axis. The position and length of homopolymers for each base is given on the x-axis to facilitate interpretation (green: A, red: T, black: G, blue: C). Click here for file

Additional file 3 **Breakdown of error rate variation using all available variables**. For each plate, we used a logistic model to decipher the role of each selected variable in explaining the variation of error rate (see materials and methods). The figure is broken down by error type: a) insertions, b) deletions, c) mismatches and d) ambiguous base calls. We tested the deviance from the complete model by breaking down the model into the sum of three terms: the first exclusive to the single effect of the variable considered (in black), the second exclusive effect of the rest of the variables without the variable of interest (in gray) and the last expressing the sum of the effects of interactions between the variable considered and the other variables (in white). The contribution of each term (the proportion) for a considered variable can be viewed on the y-axis. Additional file 3 displays the results for plates #2 and #3 (results from the plate #1 are presented as Figure 2). Click here for file

Additional file 4 **Spatial localization of error rate variation**. For each error type and the sequence length, the x-axis represents the spatial localization of 454 reads as x-coordinates and the y-axis represents the y-coordinates on the PT plate. The results presented in this additional data file 4 correspond to plates #2 and #3. The strips represent the regions. We display separately the four types of error (insertions, deletions, mismatches and ambiguous base calls) and the length of the generated sequences. Colors represent the ranges of error rates from 0 to 1 (or the length of the sequences from 0 to 500), using a sliding window (see materials and methods). Click here for file

Additional file 5 **FASTA file of the 6 reference sequences**. The six reference DNA sequences used in this analysis are found in the corresponding FASTA file. They correspond to the control DNA fragments of type I provided with 454 GS-FLX Titanium sequencing kits. As such, the polymorphism displayed by the sequences corresponds purely to sequencing errors. Click here for file

Additional file 6 **Raw data sequences from 454 GS-FLX Titanium sequencing**. This file contains three archives, including the raw FASTA files for each sequencing run. Click here for file
