# Supplementary figures and images for: Accuracy and quality assessment of 454 GS-FLX Titanium pyrosequencing
Source: BMC Genomics. 2011 May 19;12:245. doi: 10.1186/1471-2164-12-245 (PMC3116506; doi:10.1186/1471-2164-12-245)

**A** Number of sequences necessary to obtain a majority of sequences identical to the reference

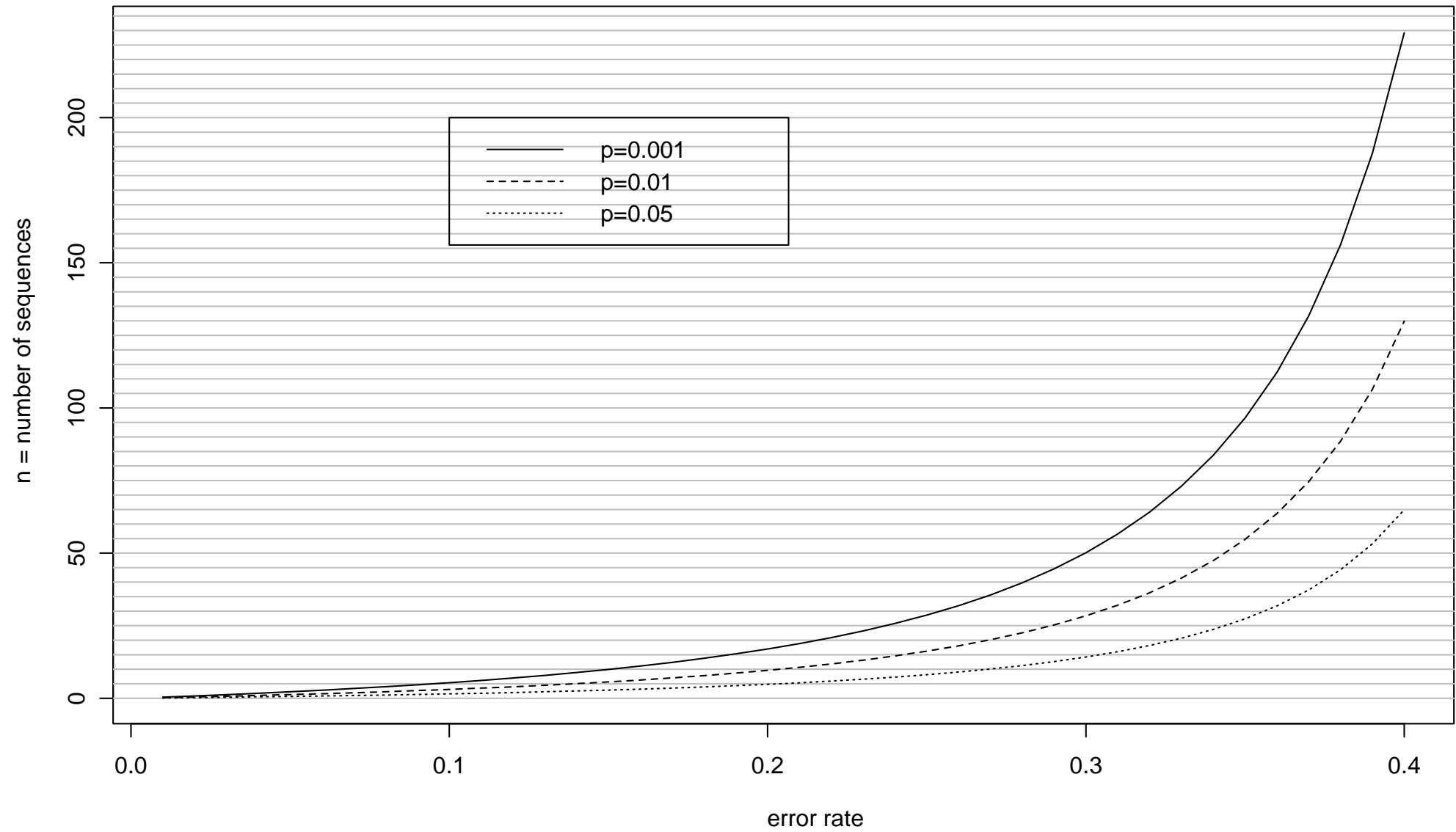

b

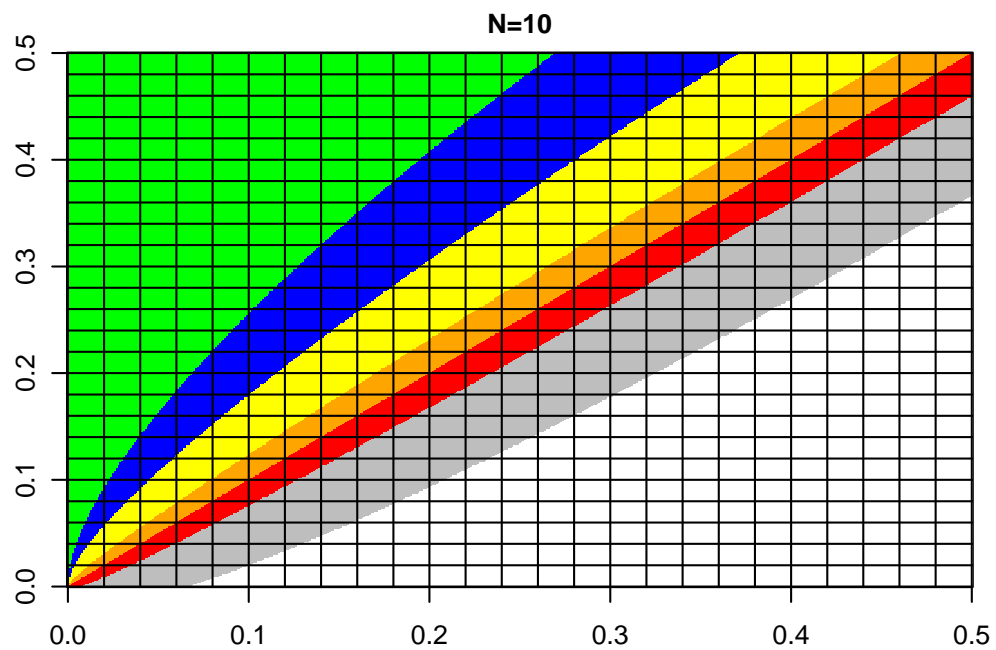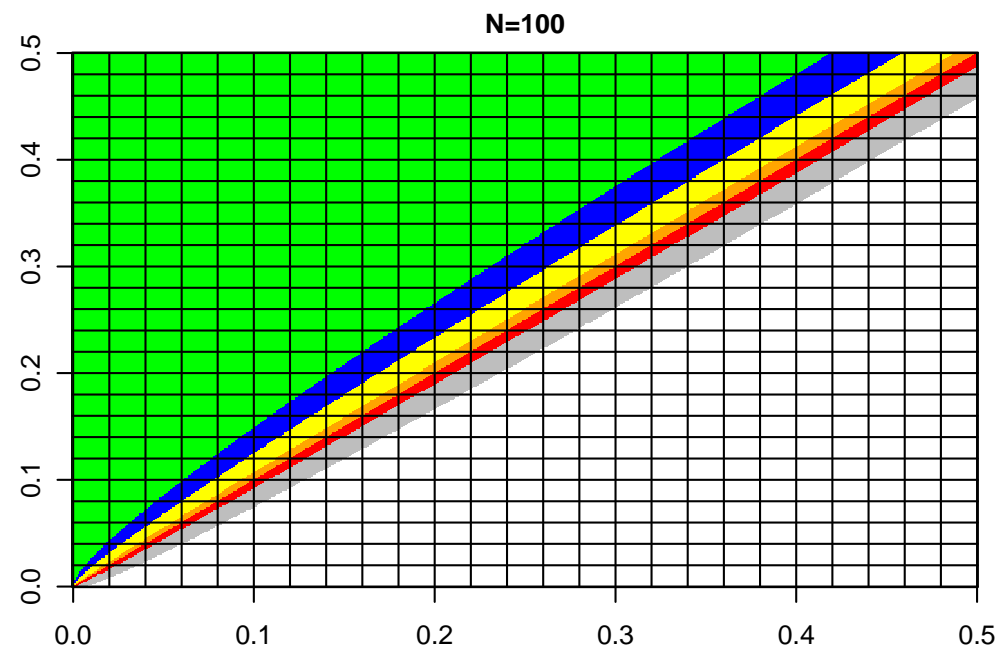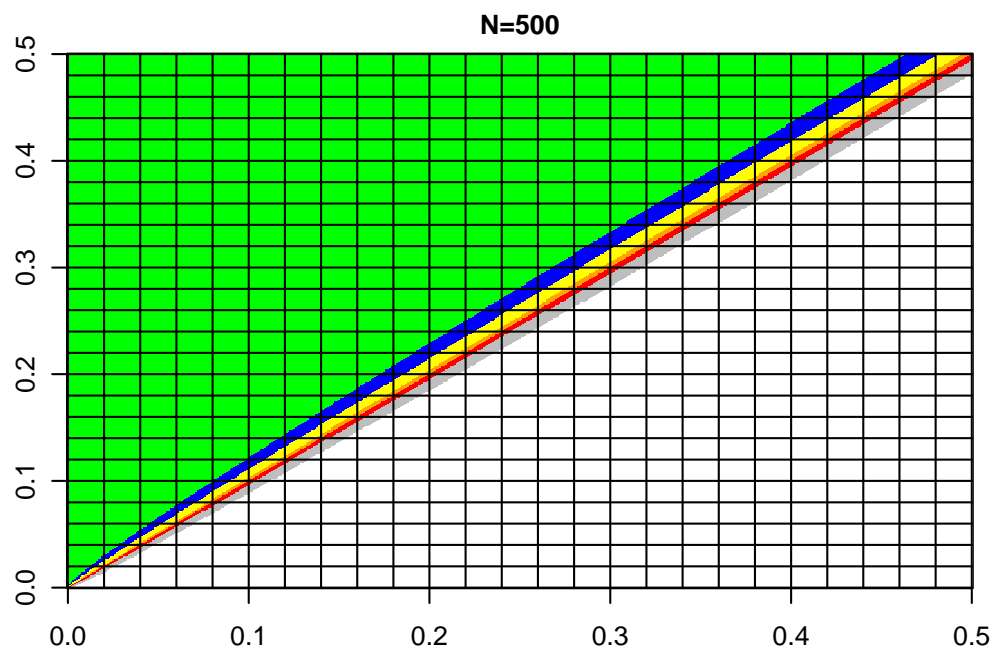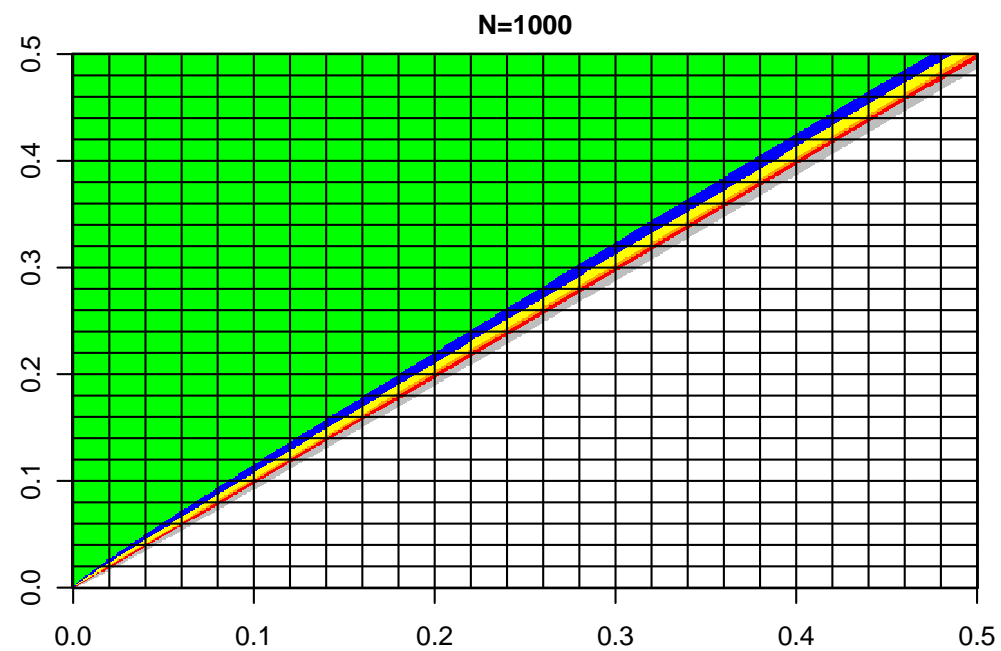

Supplement: Additional file 1 — Number of sequences to correct erroneous positions. 1a: this file illustrates the number of sequences necessary to obtain a majority of correct sequences. The x-axis shows the error rate and the y-axis shows the number of sequences needed, according to three possible probabilities: 0.001 0.01 and 0.05. 1b the x-axis shows the error rate for a given position (ranging from 0 to 0.5); the y-axis shows the cumulative proportion of erroneous sequences sampled (ranging from 0 to 0.5) in the total sample. Sample size varies from 10 to 100, 500 and 1,000 sequences. For a given error rate and a cumulative proportion of erroneous sequences in the sample of size N, the probability of observing this combination is indicated in color: green: 1 to 0.95, blue: 0.95 to 0.8, yellow: 0.8 to 0.6, orange: 0.6 to 0.5, red: 0.5 to 0.4, gray: 0.4 to 0.2 and white: below 0.2. For example, if the error rate is 0.2, the probability of observing a cumulative proportion of erroneous sequences in the sample of between 0 and 0.2 ranges between 0.4 and 0.5 (red envelope). In this case, the probability of there being 20% erroneous sequences in the sample is between 0.4 and 0.5. If we consider the same error rate (0.2) with 40% erroneous sequences, then the probability ranges from 0.8 to 0.95 (blue envelope). If N increases, the variance of the probability envelopes decreases. [file 1471-2164-12-245-S1.PDF]

## Sequence 1

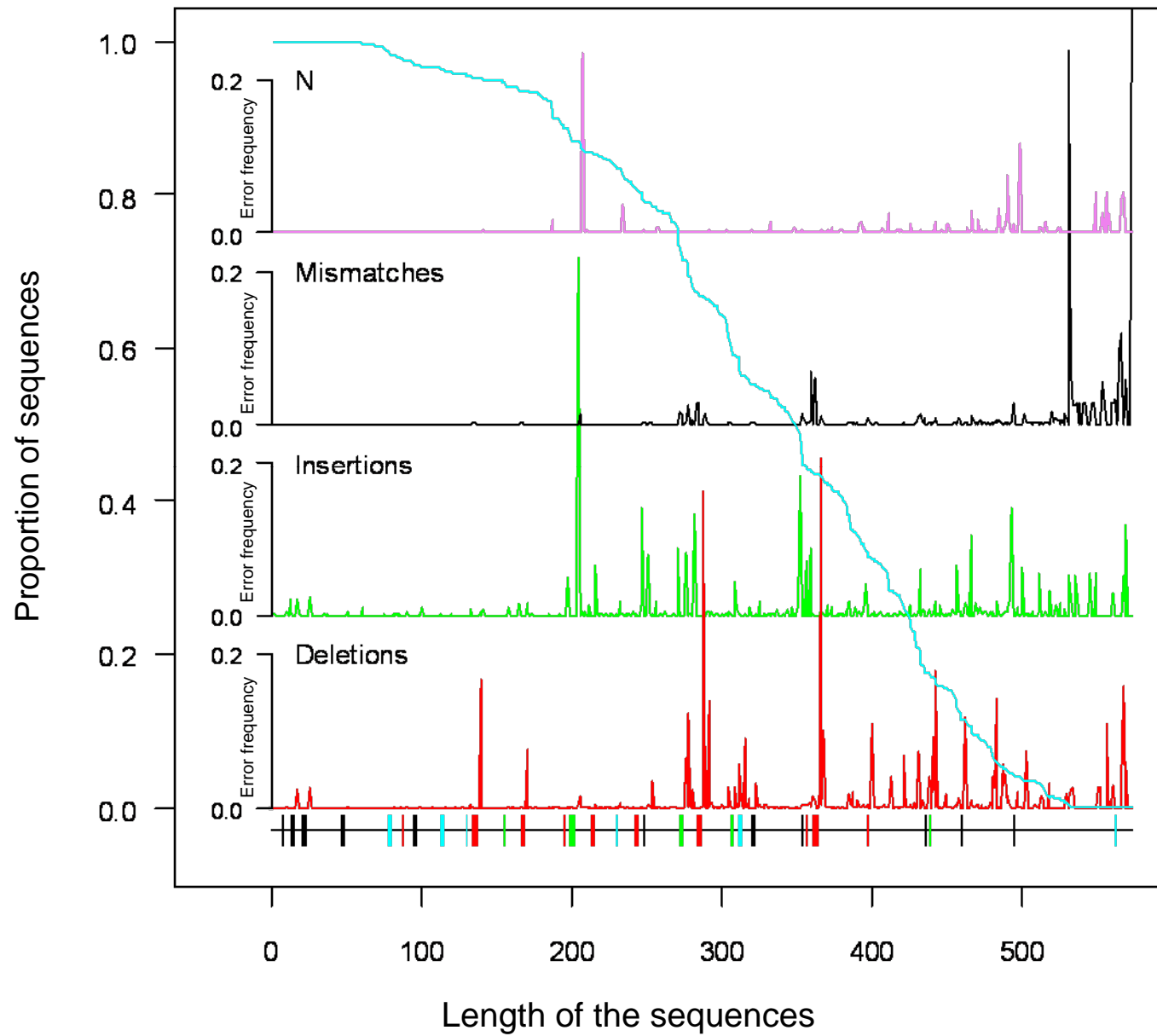

## Sequence 2

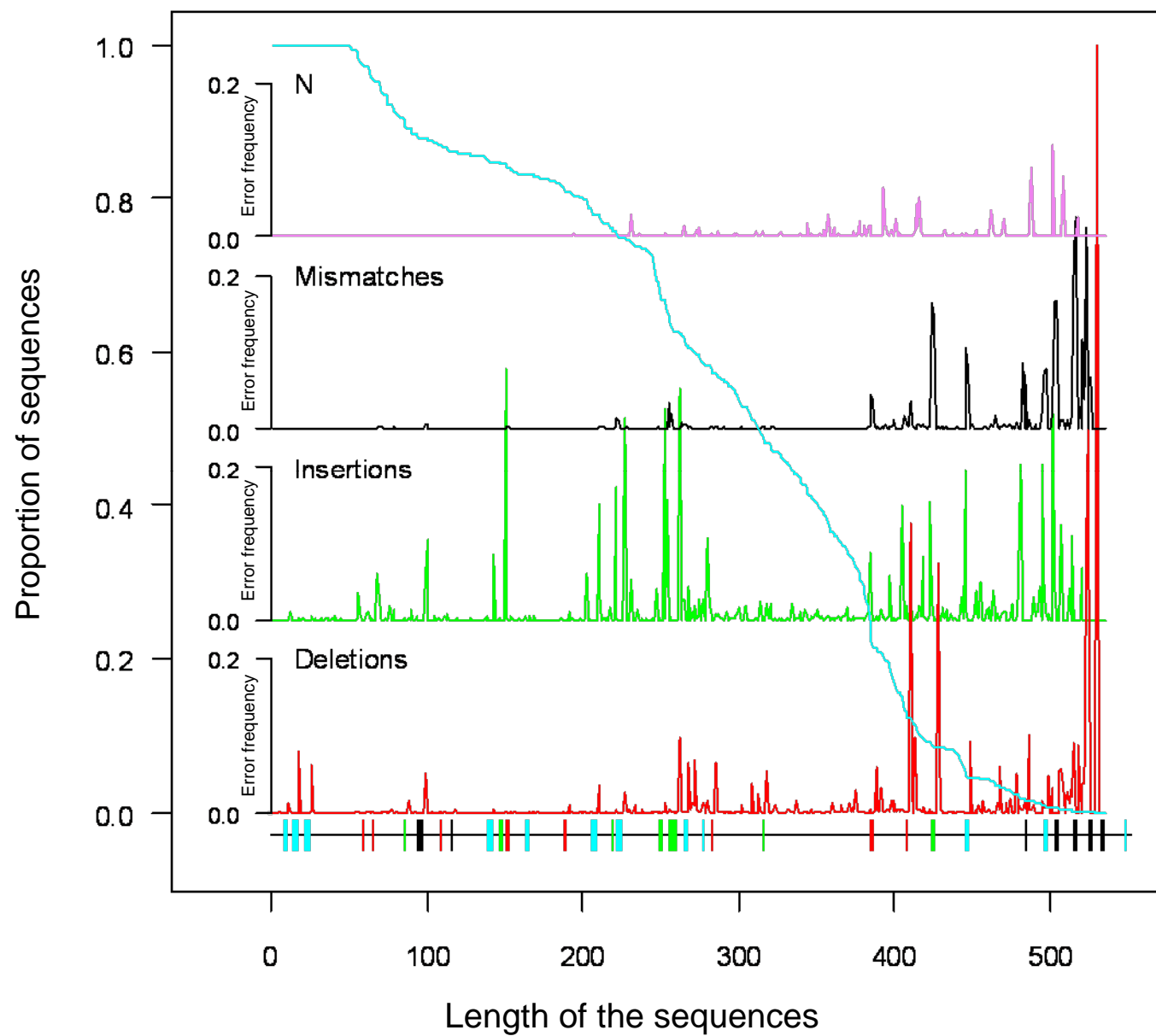

Sequence 4

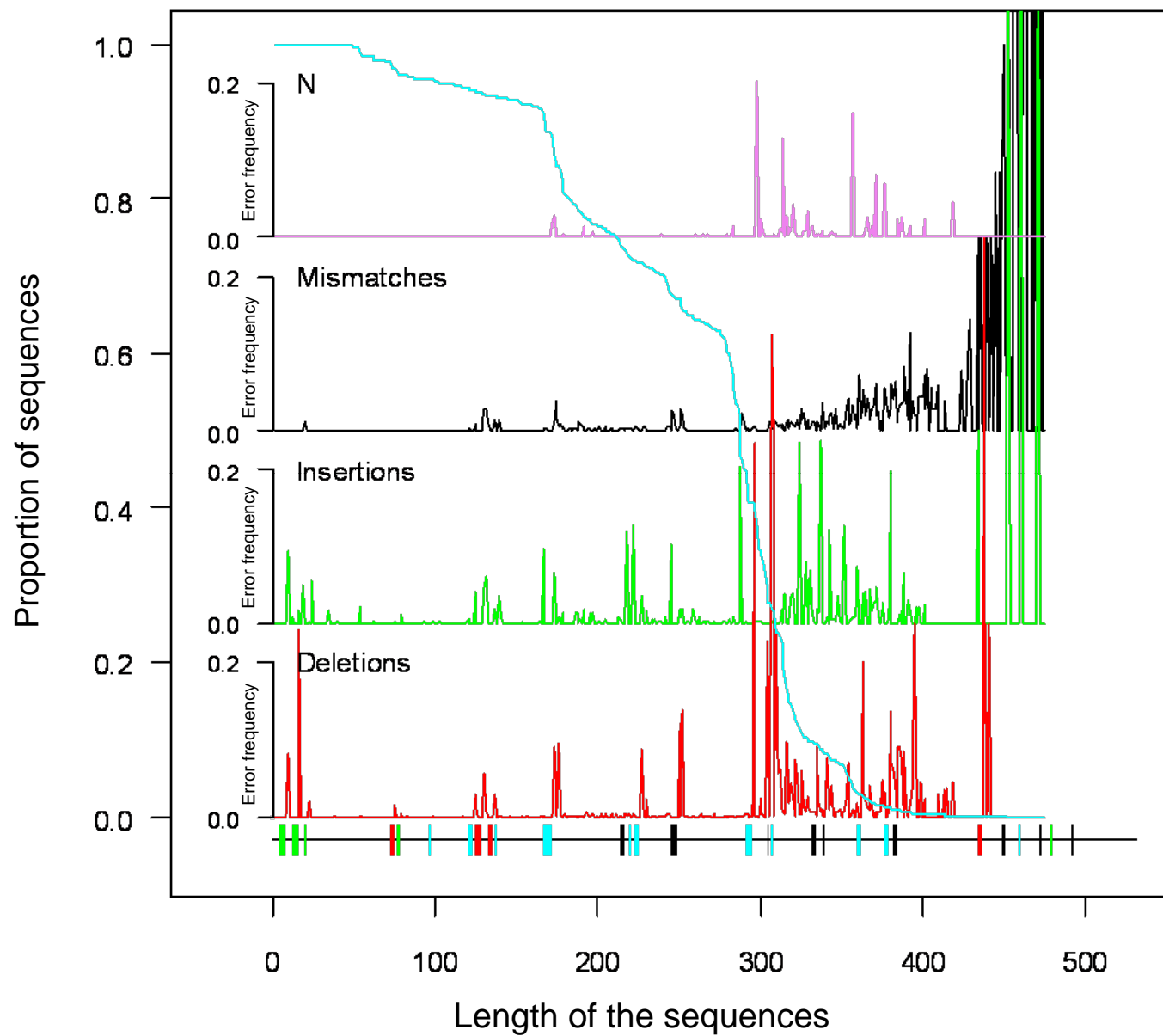

## Sequence 5

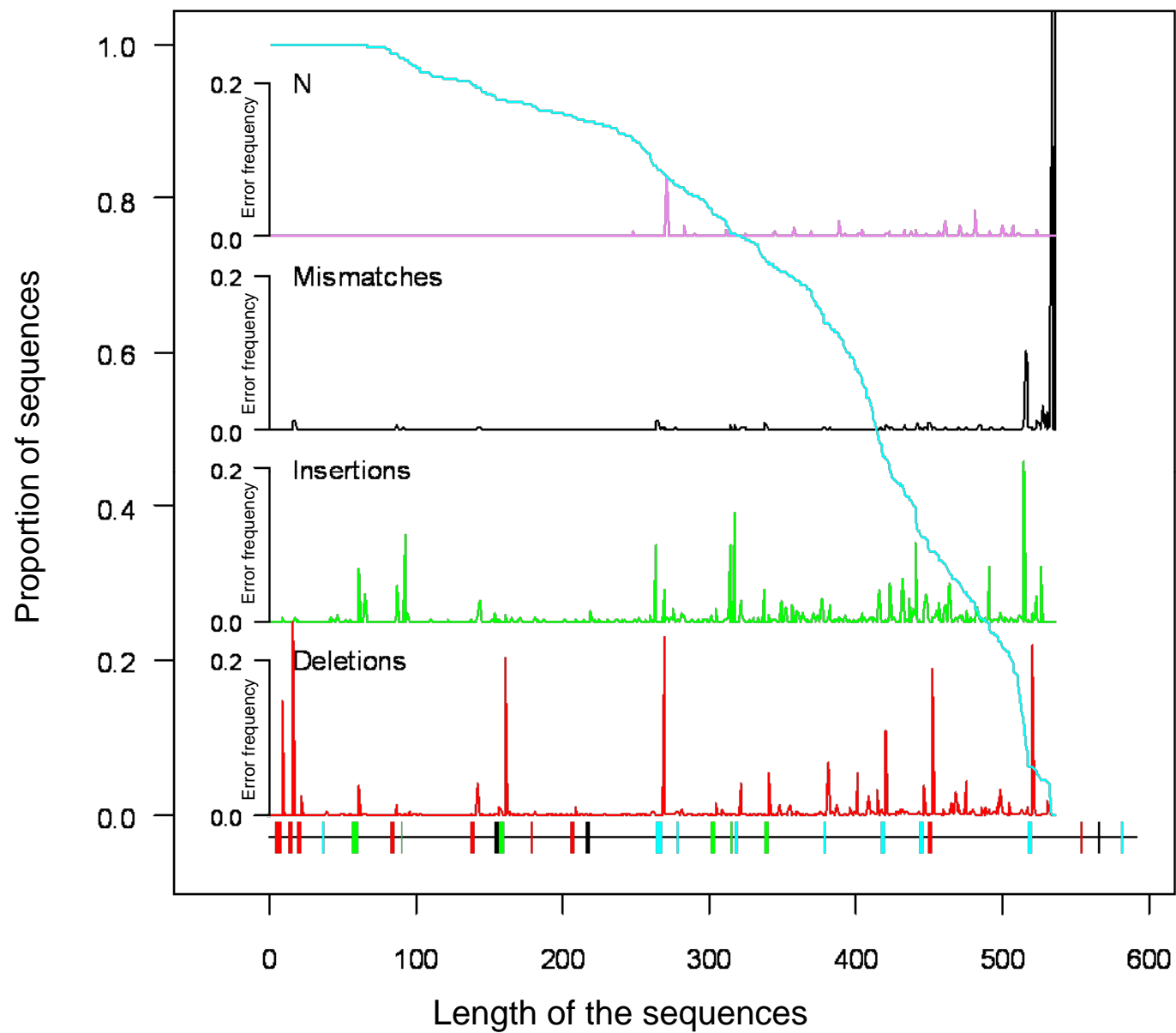

Sequence 6

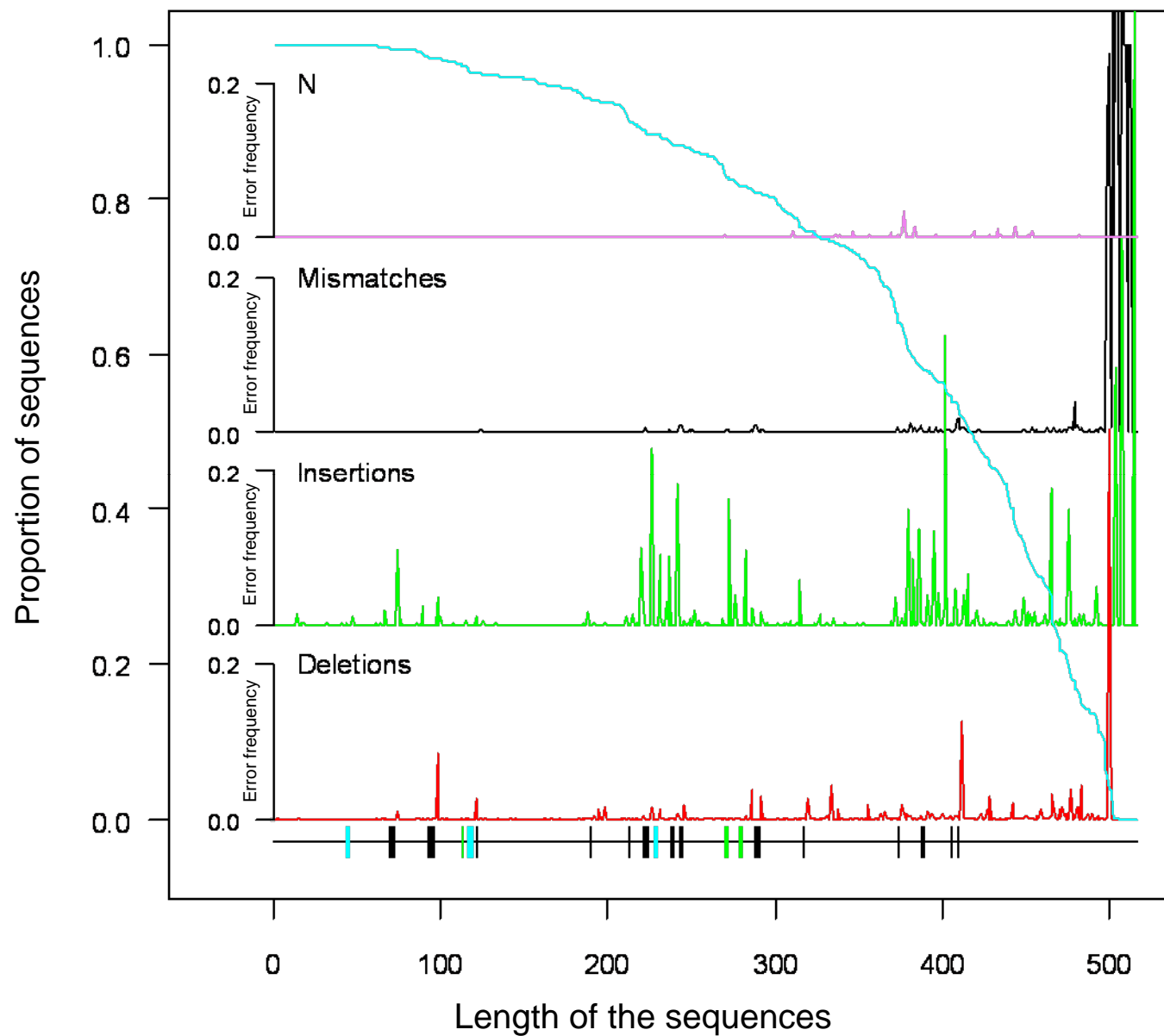

Supplement: Additional file 2 — Distribution of errors along the reference sequences. The blue line represents the proportion of sequences generated (y-axis) according to the sequence position (x-axis), using data obtained from the analysis of reference 5 reference sequences (excluding reference #3, which is displayed in Figure 1). The error rate for each type of error (insertions, deletions, mismatches and ambiguous base calls) is presented as a function of the sequence position (x-axis) and specific position on the y-axis. The position and length of homopolymers for each base is given on the x-axis to facilitate interpretation (green: A, red: T, black: G, blue: C). [file 1471-2164-12-245-S2.PDF]

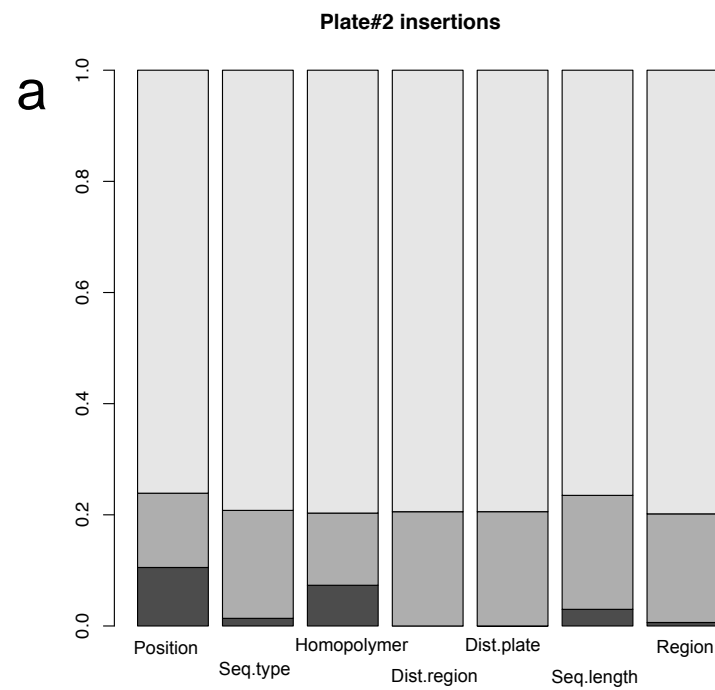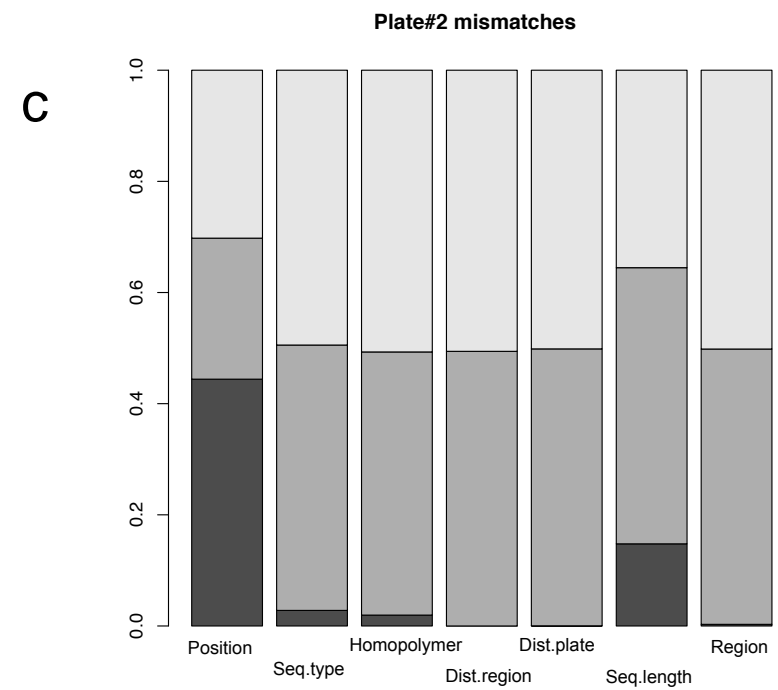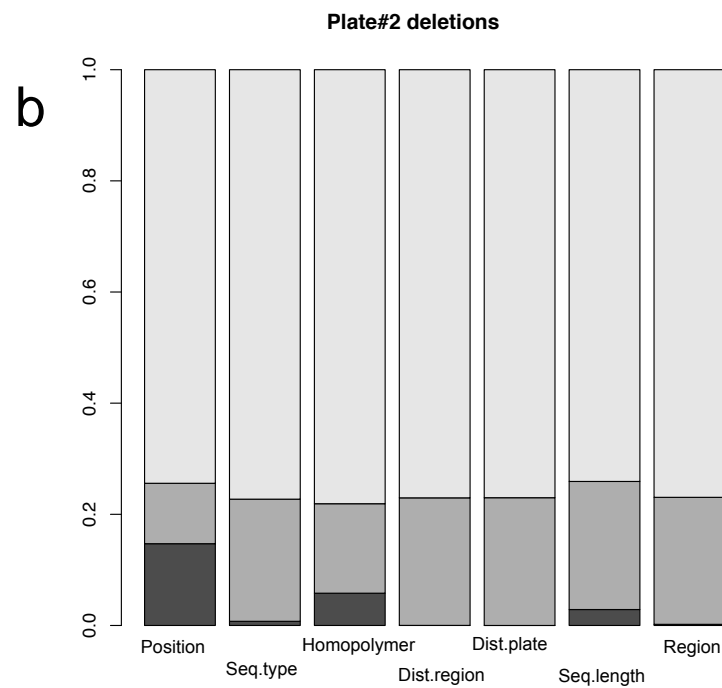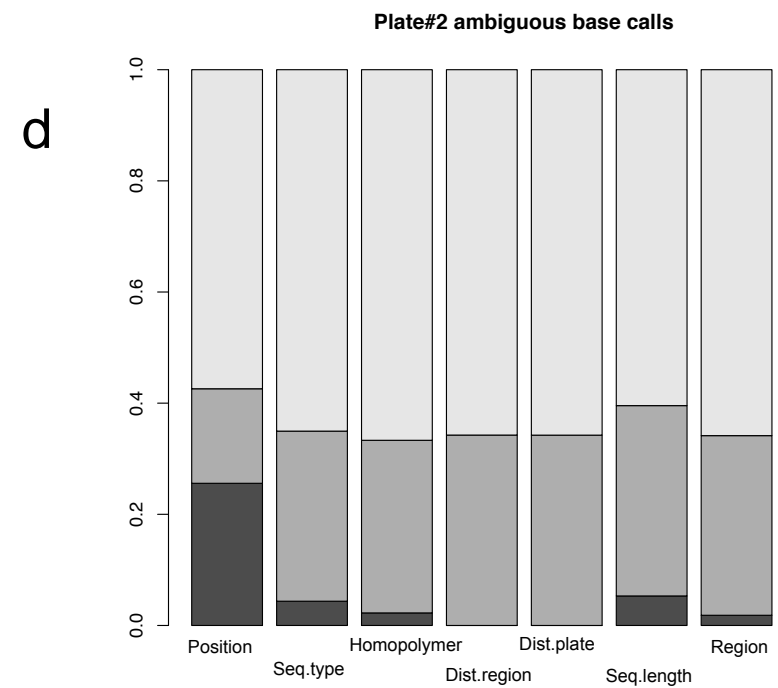

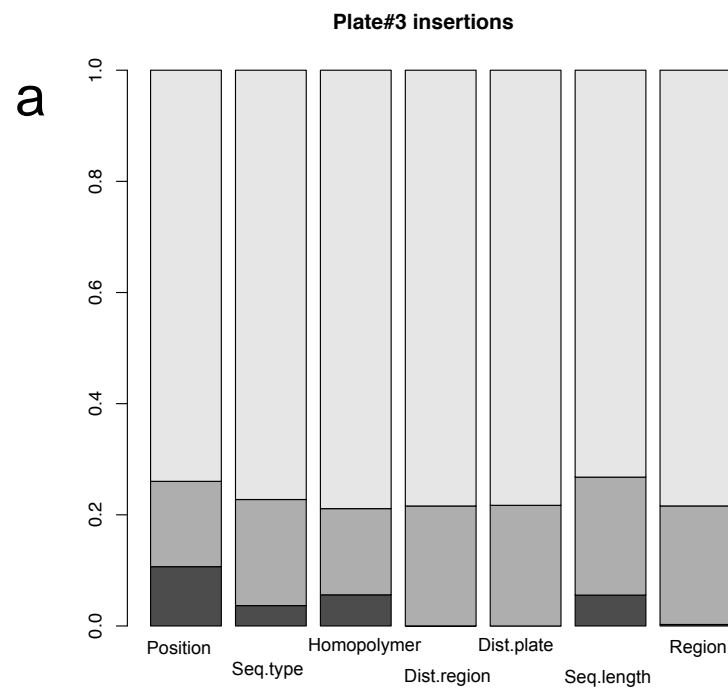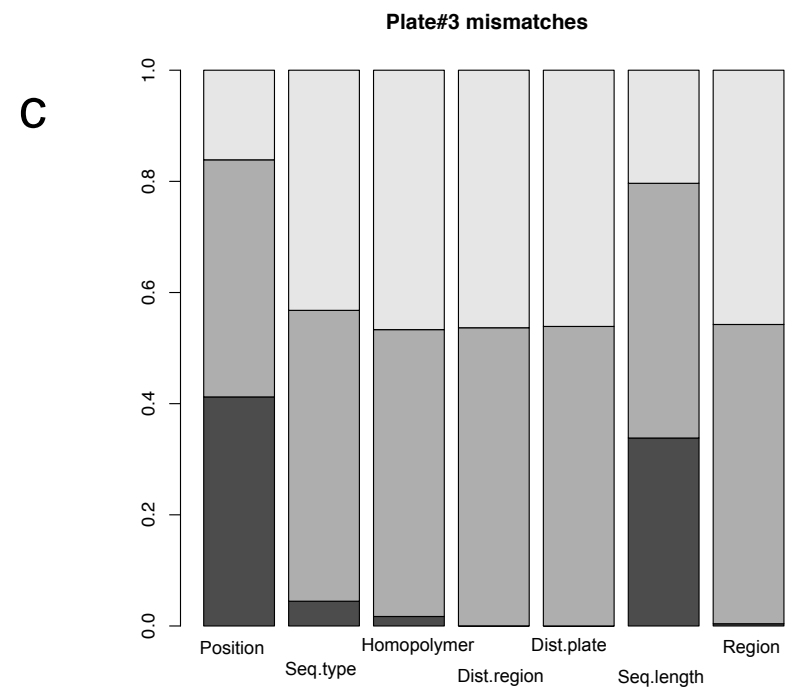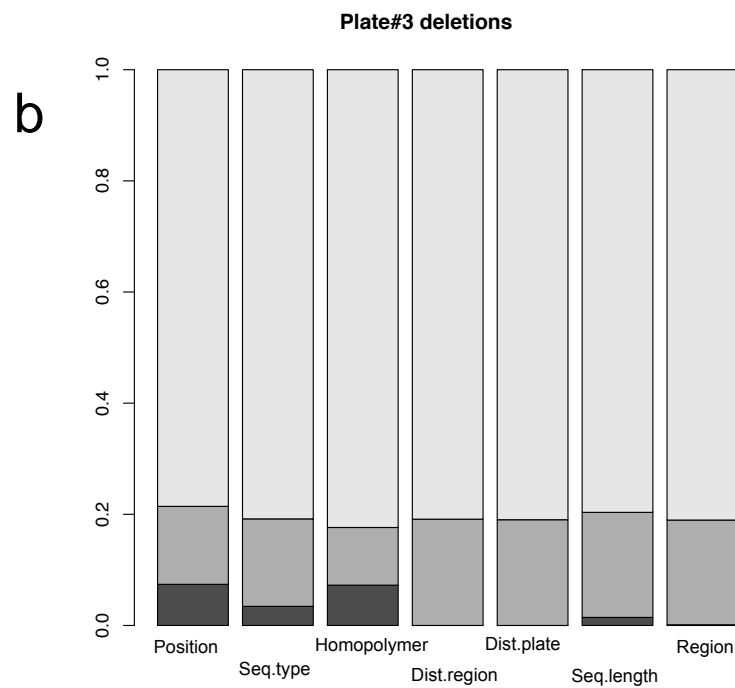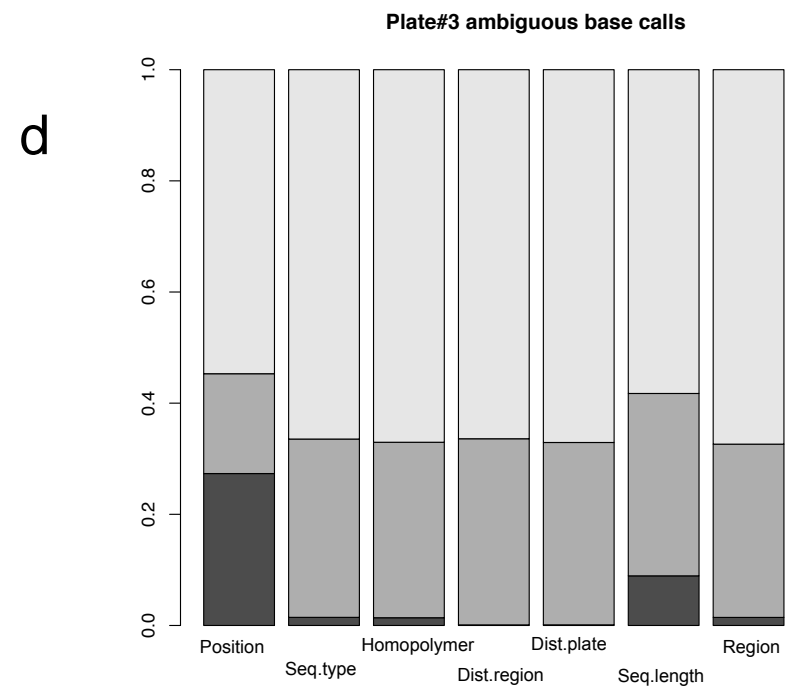

Supplement: Additional file 3 — Breakdown of error rate variation using all available variables. For each plate, we used a logistic model to decipher the role of each selected variable in explaining the variation of error rate (see materials and methods). The figure is broken down by error type: a) insertions, b) deletions, c) mismatches and d) ambiguous base calls. We tested the deviance from the complete model by breaking down the model into the sum of three terms: the first exclusive to the single effect of the variable considered (in black), the second exclusive effect of the rest of the variables without the variable of interest (in gray) and the last expressing the sum of the effects of interactions between the variable considered and the other variables (in white). The contribution of each term (the proportion) for a considered variable can be viewed on the y-axis. Additional file 3 displays the results for plates #2 and #3 (results from the plate #1 are presented as Figure 2). [file 1471-2164-12-245-S3.PDF]
