# Supplementary material for: Accuracy and quality assessment of 454 GS-FLX Titanium pyrosequencing
Source: BMC Genomics. 2011 May 19;12:245. doi: 10.1186/1471-2164-12-245 (PMC3116506; doi:10.1186/1471-2164-12-245)
Supplement: Additional file 4 — Spatial localization of error rate variation. For each error type and the sequence length, the x-axis represents the spatial localization of 454 reads as x-coordinates and the y-axis represents the y-coordinates on the PT plate. The results presented in this additional data file 4 correspond to plates #2 and #3. The strips represent the regions. We display separately the four types of error (insertions, deletions, mismatches and ambiguous base calls) and the length of the generated sequences. Colors represent the ranges of error rates from 0 to 1 (or the length of the sequences from 0 to 500), using a sliding window (see materials and methods). [file 1471-2164-12-245-S4.PDF]

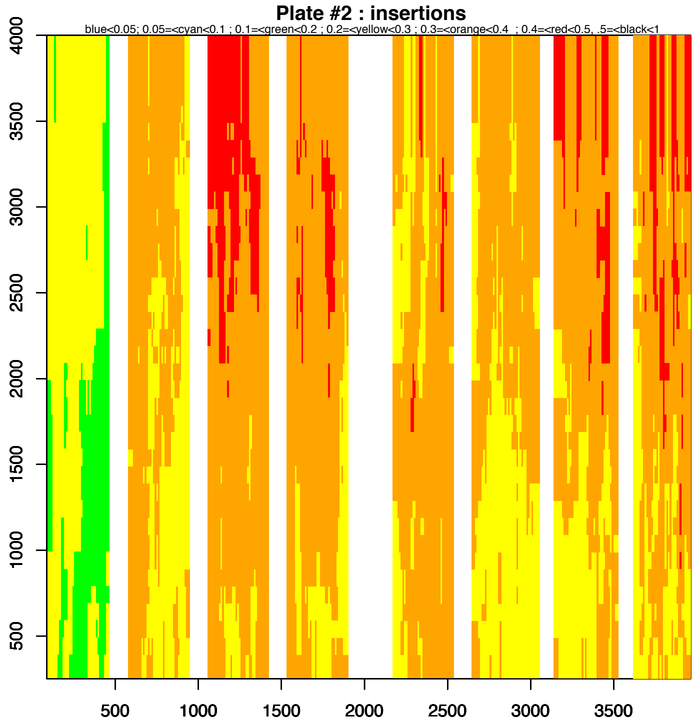
Plate #2 : insertions
blue<0.05; 0.05=<cyan<0.1 ; 0.1=<green<0.2 ; 0.2=<yellow<0.3 ; 0.3=<orange<0.4 ; 0.4=<red<0.5, .5=<black<1
500
1000
1500
2000
2500
3000
3500
4000

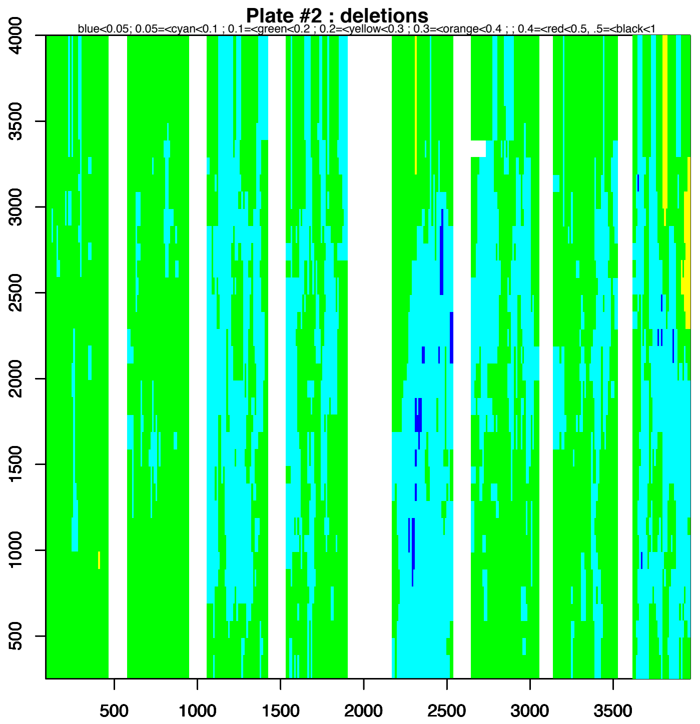
Plate #2 : deletions
blue<0.05; 0.05=<cyan<0.1 ; 0.1=<green<0.2 ; 0.2=<yellow<0.3 ; 0.3=<orange<0.4 ; ; 0.4=<red<0.5, .5=<black<1
500
1000
1500
2000
2500
3000
3500
4000

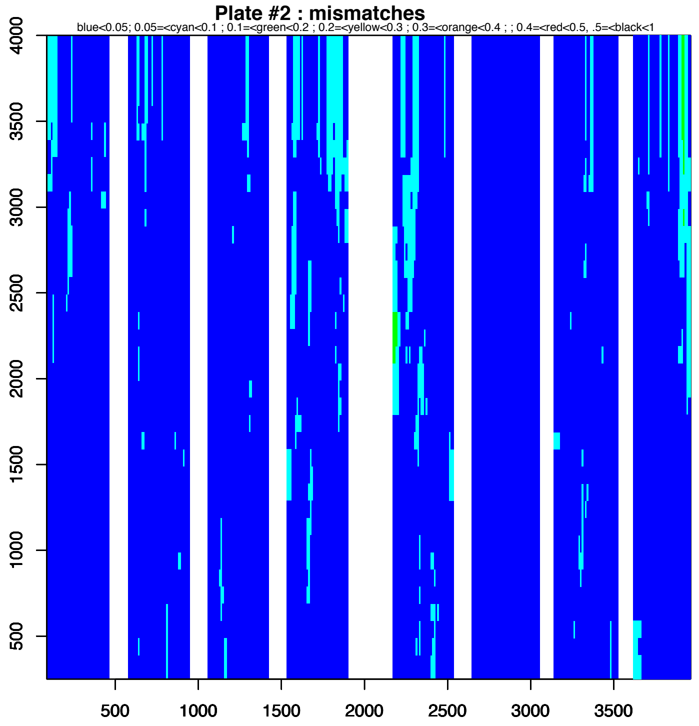
Plate #2 : mismatches
blue<0.05; 0.05=<cyan<0.1 ; 0.1=<green<0.2 ; 0.2=<yellow<0.3 ; 0.3=<orange<0.4 ; ; 0.4=<red<0.5, .5=<black<1
500
1000
1500
2000
2500
3000
3500
4000

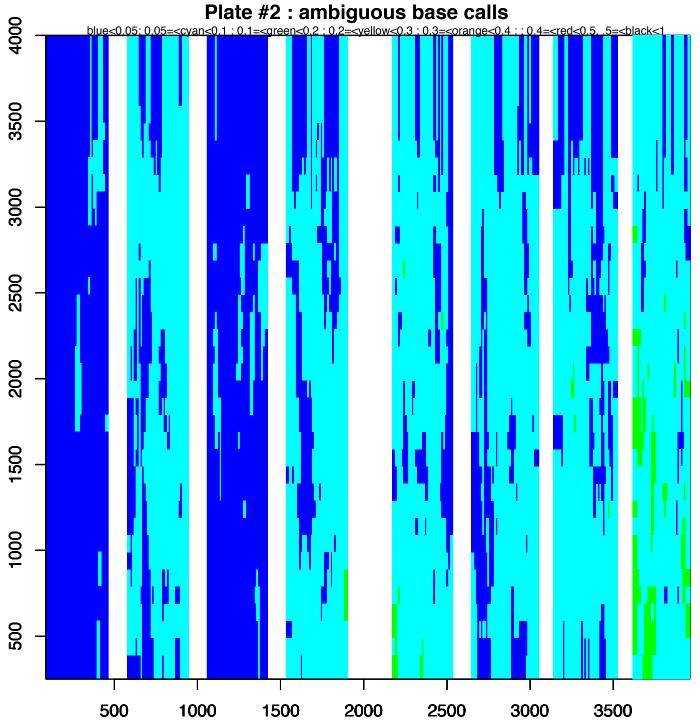
Plate #2 : ambiguous base calls
blue<0.05; 0.05=<cyan<0.1 ; 0.1=<green<0.2 ; 0.2=<yellow<0.3 ; 0.3=<orange<0.4 ; ; 0.4=<red<0.5, .5=<black<1
500
1000
1500
2000
2500
3000
3500
4000

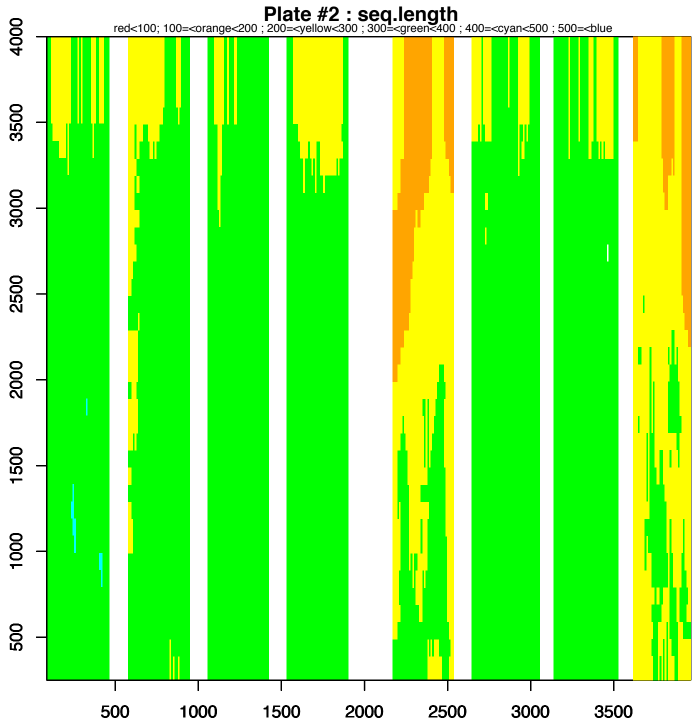
Plate #2 : seq.length
red<100; 100=<orange<200 ; 200=<yellow<300 ; 300=<green<400 ; 400=<cyan<500 ; 500=<blue
500
1000
1500
2000
2500
3000
3500
4000

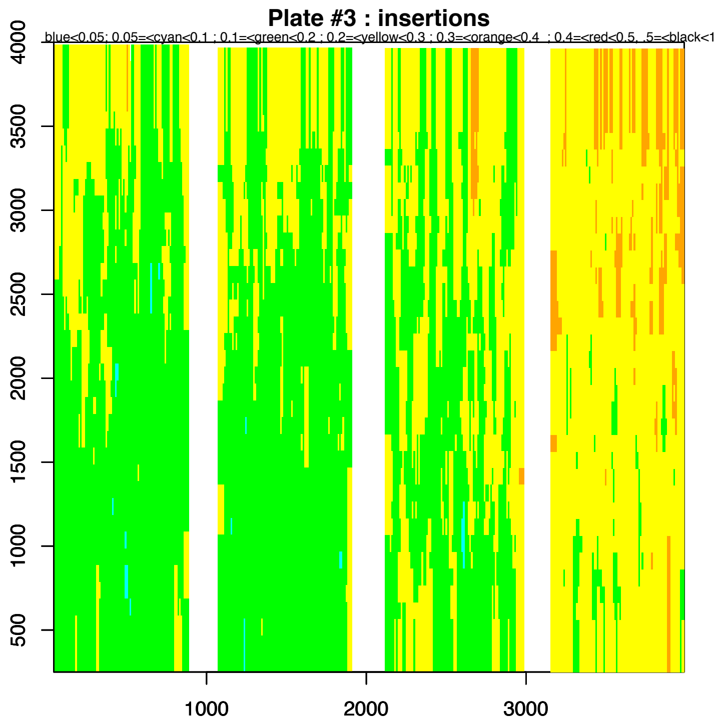
Plate #3 : insertions
blue<0.05; 0.05=<cyan<0.1 ; 0.1=<green<0.2 ; 0.2=<yellow<0.3 ; 0.3=<orange<0.4 ; 0.4=<red<0.5, .5=<black<1
500
1000
1500
2000
2500
3000
3500
4000
1000
2000
3000

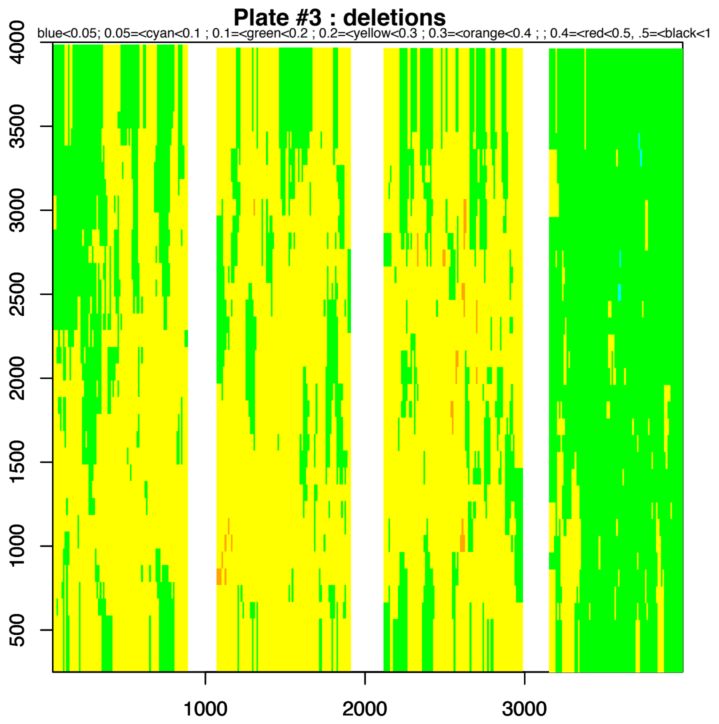
Plate #3 : deletions
blue<0.05; 0.05=<cyan<0.1 ; 0.1=<green<0.2 ; 0.2=<yellow<0.3 ; 0.3=<orange<0.4 ; ; 0.4=<red<0.5, .5=<black<1
500
1000
1500
2000
2500
3000
3500
4000
1000
2000
3000

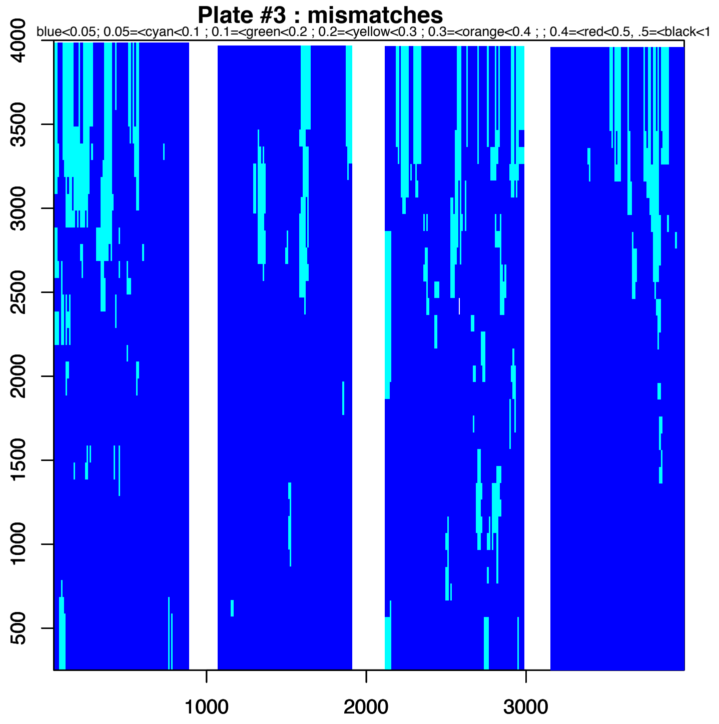
Plate #3 : mismatches
blue<0.05; 0.05=<cyan<0.1 ; 0.1=<green<0.2 ; 0.2=<yellow<0.3 ; 0.3=<orange<0.4 ; ; 0.4=<red<0.5, .5=<black<1
500
1000
1500
2000
2500
3000
3500
4000
1000
2000
3000

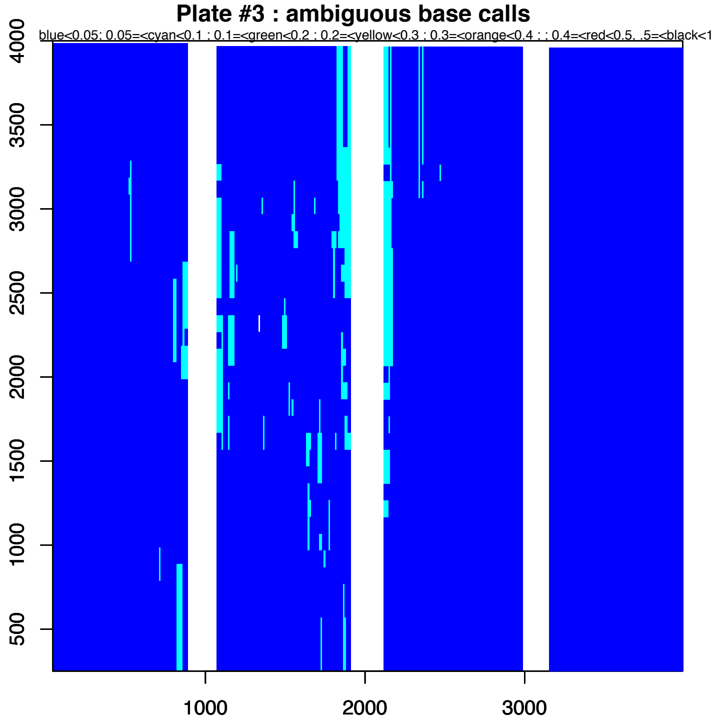
Plate #3 : ambiguous base calls
blue<0.05; 0.05=<cyan<0.1 ; 0.1=<green<0.2 ; 0.2=<yellow<0.3 ; 0.3=<orange<0.4 ; ; 0.4=<red<0.5, .5=<black<1
500
1000
1500
2000
2500
3000
3500
4000
1000
2000
3000

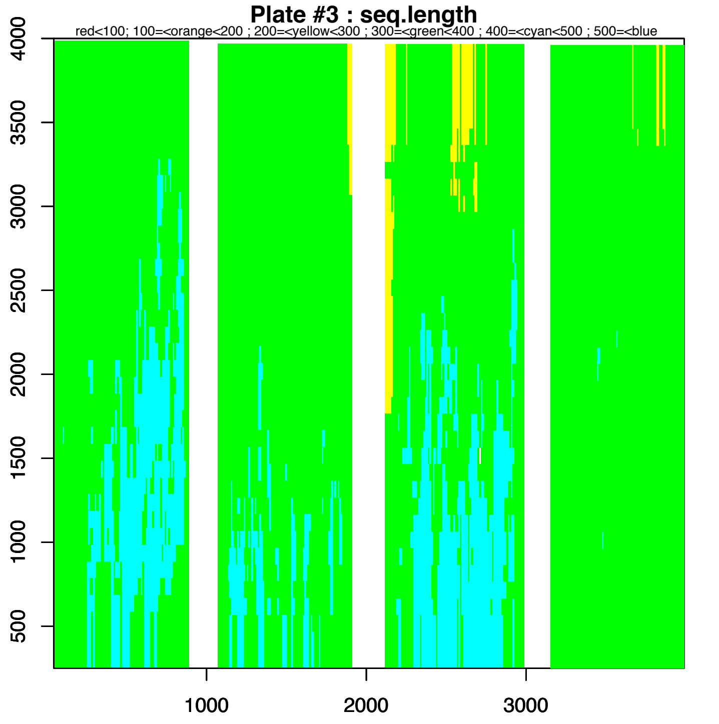
Plate #3 : seq.length
red<100; 100=<orange<200 ; 200=<yellow<300 ; 300=<green<400 ; 400=<cyan<500 ; 500=<blue
500
1000
1500
2000
2500
3000
3500
4000
1000
2000
3000
